# Measurement and Documentation of Physical Health Parameters of Patients With a Diagnosis of an Eating Disorder at the Cove (Inpatient Unit), in Accordance With the MEED Guidelines

**DOI:** 10.1192/bjo.2025.10612

**Published:** 2025-06-20

**Authors:** Iffath Javeed, Lin John, Amith Paramel, Sameer Alji-Mohamed

**Affiliations:** Lancashire and South Cumbria NHS Foundation Trust, Preston, United Kingdom

## Abstract

**Aims:** Over the recent years hospital admissions for eating disorders have been on the rise and RCPsych identified this is partly attributable to lack of guidance and training amongst healthcare professionals in recognition of the, often missed, alarming signs.

The Medical Emergencies in Eating Disorders guidelines (MEED) have been introduced to enable assessment and risk stratification of patients with an eating disorder based on a number of physical health parameters to aid emergency management. The complex interplay between physical and mental health of eating disorder patients highlights the importance of good documentation and assessment of clinical factors which would help in seeking appropriate specialist input.

The aim of the audit is to determine if young people admitted to The Cove with a diagnosis of eating disorder have clear documentation on their notes which include physical health parameters in accordance with MEED.

**Methods:** Data was collected retrospectively from electronic notes of service users with a diagnosis of eating disorder (n=20) admitted to a CAMHS unit over a 30-month period. This baseline audit addresses documentation of evidence of physical health parameters.

**Results:** The baseline audit focused on documentation of physical health parameters during the period of admission. A high assurance of 80% and above was recorded for: weight for height, heart rate, ECG and blood investigations at The Cove during this audit cycle. A limited assurance whereby the compliance was 70–75% was noted for monitoring of core temperature. There was some underperformance, such as, in documentation of SUSS test and/or hydration status.

**Conclusion:** The baseline audit achieved an overall compliance of 69%, providing not a high assurance in the monitoring and documentation of physical health parameters on the electronic notes. The compliance calculations were based on a small cohort of service users.

The MDT would need to consider implementing a template that would cover the parameters expected by the MEED guidelines. Following implementation of the tool a re-audit would be performed in due course.

